# Study of cytomegalovirus infection in idiopathic infertility men referred to Shariati hospital, Tehran, Iran

**Published:** 2014-02

**Authors:** Masoud Habibi, Alireza Bahrami, Afsaneh Morteza, Mohammad Ali Sadighi Gilani, Gholamreza Hassanzadeh, Mohsen Ghadami, Hamid Choobineh

**Affiliations:** 1 Iranian Center for Breast Cancer, Academic Center for Education Culture and Research (ACECR), Tehran, Iran.; 2 Tehran University of Medical Sciences, Tehran, Iran.; 3 Department of Urology, Tehran University of Medical Sciences, Tehran, Iran.; 4 Department of Anatomy and Reproductive Biology, Tehran University of Medical Sciences, Tehran, Iran.; 5 Department of Medical Genetics, Endocrine and Metabolism Research Institute, Tehran University of Medical Sciences, Tehran, Iran.; 6 School of Allied Medical Sciences, Tehran University of Medical Sciences, Tehran, Iran.; 7 Zoonosis Research Center, Tehran University of Medical Sciences, Tehran, Iran.

**Keywords:** *Male infertility*, *Cytomegalovirus infection*, *Sperm morphology*

## Abstract

**Background:** Cytomegalovirus (CMV) is a prevalent infection in humans. Recent studies have shown the role of CMV infection in male infertility disorder.

**Aim:** Here we aimed to study the role of CMV infection in men with idiopathic infertility.

**Materials and Methods: **We performed a case-control study of CMV serology in 200 patients attending male infertility clinic of a university hospital. There were 154 men diagnosed with infertility and 46 men without infertility. The patients were asked to donate their sperm, blood, and urine. The presence of CMV infection was studied using quantitative polymerase chain reaction.

**Results:** CMV infection was present in 25 of all the studied participants. Controls had a higher sperm count and sperm motility and sperm morphology compared to patients. There were no significant differences in the studied variables between those with and without CMV infection, nor in patients, neither in controls. Sperm morphology was negatively correlated with cigarette smoking (r=-0.15; p<0.03). Even though the prevalence of CMV infection was higher in patients with infertility in control and patient (5/46 vs. 20/154) respectively, this was not statistically significant.

**Conclusion:** We did not show a significant role for CMV infection in male infertility. Based on the previous studies, it could be assumed that CMV infection is an important part of the male infertility and its treatment would improve the sperm quality, however this was not confirmed by the present study.

## Introduction

Infertility primarily refers to the biological inability of a person to contribute to conception. Male infertility refers to the inability of a male to cause pregnancy in a fertile female. In humans it accounts for 40-50% of infertility problems (-). Male infertility is commonly due to deficiencies in the semen, and semen quality is used as a surrogate measure of male fecundity (4). Recent studies have shown the role of viral infections as an idiopathic pathogenesis of male infertility (3, -). Cytomegalovirus (CMV) is a viral genus of the viral family known as herpes viridae or herpes viruses. The one that infects humans is known as human CMV or human herpesvirus-5 (HHV-5), and is the most studied of all CMV’s. They are frequently associated with the salivary glands in humans and other mammals. 

Human CMV infection is typically unnoticed in healthy people, but can be life-threatening for the immune-compromised patients, such as HIV-infected persons, organ transplants recipients, or newborn infants. After infection, CMV persists in the host because the viral genome encodes multiple proteins that interfere with immune reaction and antigen presentation. Recent studies have shown that it play a significant role in male infertility, and its early detection by the nested polymerase chain reaction (PCR) technique will permit successful antiviral therapy to increase the possibility for fertility restoration. (5). Here we aimed to study the impact of CMV infection detected by quantitative PCR in patients attending male infertility clinic in Iran. 

## Materials and methods

We performed a cross sectional study of the presence of CMV measured by quantitative PCR in semen, blood, and urine of 154 men with the diagnosis of infertility attending male infertility clinic of a university hospital and 46 age, and BMI matched controls. Inclusion criteria: 1. satisfaction of patient’s refers to center for infertility in shariati hospital in Tehran, Iran. 2. Men should be the cause of infertility. 3. Extra speciments should be taken from patients. Exclusion criteria: if the patients did not have the above inclusion criteria. Infertility was diagnosed according to the criteria of the American Society for Reproductive Medicine (8).

All the patients were diagnosed in a month and were not on any treatment. Age less than 18 years old and any type of malignant disorders was considered as exclusion criteria. Demographic data including age, sex, duration of infertility, job, cigarette smoking, history of any other disease and surgery was investigated in all participants. All patients underwent sperm, blood and urine sampling. Quantitative PCR was used to measure the presence of CMV in the urine, blood, and semen. 

The research was carried out according to the principles of declaration of Helsinki. The local ethics review committee of Tehran University of Medical Sciences approved the study protocols. All participants gave written informed consent before participation. 

**Samples**

The blood, urine, and semen samples were collected in the morning. All the samples were prepared and were kept at -70^o^C until analysis. The blood was clotted centrifuges and the cells were kept at -70^o^C until analysis.

**Urine concentration protocol**

A total of 10 ml of urine was divided into 5 micro tubes with the volume of 2 ml. The tubes were centrifuged for 10 min with 5800 rpm. The supernatant was removed and all the tubes were refilled with phosphate-buffered saline (PBS). The tubes were centrifuged for 10 min with 5800 rpm. The supernatant was removed and the precipitant was removed with 40 µl of PBS. The material in all the tubes was added together. 

**Semen Preparation protocol**

A total of 250 µl of semen with 10 ml of buffer number 1 (150 mM NaCl, 10 mM EDTA pH= 8.0), were added to the cortex tube. The tubes were vortex for 10 seconds. The tubes were centrifuges for 10 min at 4000 rpm. The supernatant was carefully removed (1 ml remained in the tube). The tubes were vortex for 10 sec. The material in the tubes was added to a new tube. The tubes were centrifuges for 2 min with the highest speed. The supernatant was removed and 300µl of buffer number 2 was added to the tubes. Buffer 2: (100 mM Tris. C, 10 mM EDTA, 500 mM NaCl, 1% SDS, 2% β. Mercaptoethanol; pH= 8.0). 

**PCR techniques**

DNA was extracted from the samples using the DNA assay tissue kit (QIAGEN, Valencia, CA) according to manufacturer’s protocol. Extracted DNA was subjected to PCR using the following primers: 1) 5’-AAGGGACTGG CTGCTATTG-3’, 2) 5’- AGAAAAGCGGCCA TTTTC-3’ set for mutated allele and 3) 5’- AGTTCAATGGCGTTCCG-3’, 4) 5’-CATGTCA GTAGTACATTAGAG-3’ set for normal allele. RED Taq Ready Mix PCR reaction containing 0.4 mM deoxynucleo side triphosphates (dNTPs), 20 mM Tris-HCL, pH=8.3, with 100 mM KCl, 3mM MgCl_2_, 0.002% gelatin, stabilizers and 0.06 IU/µl *Taq* polymerase (Sigma Aldrich, St Louis, MO) was used for PCR amplification in a total volume of 50 µl of reaction containing 100 ng template DNA and 150 ng primers.

PCR condition was set as a denaturation at 94^o^C for 5 min, followed by 30 cycles of 94^o^C for 45 sec, 56^o^C for 45 sec, 72^o^C for 1 min, and final extension at 72^o^C for 10 min. PCR products were subjected to electrophoresis in 1% agarose gel and visualized under UV light. Sperm count; sperm motility and sperm morphology was measured for all participants, by a single technician trained for these measurements, using microscopic evaluation. The technician was blind to the cases and controls.

The presence of CMV was demonstrated by quantitative PCR according to the protocols of Tehran University of Medical Sciences. 

**Statistical analysis**

The statistical package SPSS 17 for windows (Chicago, Illinois, USA), was used for analysis. Variables distributed normally are presented as mean and standard error of mean (SEM). To compare the prevalence of CMV infection between groups, Chi square analysis was employed. Significance was set at a p-value lower than 0.05. Spearman correlation coefficient was employed to study the correlation between the studied variables. 

## Results

There were 25 patients with CMV infection, 22 of them had one site of infection and 3 of them had 2 sites of infection. Even though the prevalence of CMV infection was higher in patients with infertility in control and patient (5/46 vs. 20/154) respectively,(p=0.12), this was not statistically significant. Characteristics of the participants are presented in table I. 

There were 154 cases and 46 controls. Patients in the control group had a higher sperm count (30.4±2.1 vs. 21.7±1.5, p<0.001) and sperm morphology (45.1±1.8 vs. 15.8±1.1, p<0.001) and sperm motility (23.9±2.0 vs. 19.5±1.0, p<0.05) compared to patients with infertility. There were no significant differences between cases and controls in other studied variables (Table I). The mean sperm motility, sperm count and sperm morphology was not different between patients with and without CMV infection (Table II). 

Sperm morphology was negatively correlated with cigarette smoking (r= -0.15; p<0.05).

**Table I:** Presenting the primary characteristics of the participants

		**Control (n=46)**	**Infertility (n=154)**	**p-value**
Age (years)		35.00 ± 0.905	33.87 ± 0.44	.07
BMI (kg/m^2^)		23.93 ± 0.52	24.23 ± 0.34	.09
With CMV infection		41	134	.10
Without CMV infection		5	20	.08
CMV in sperm		1	12	.09
CMV in blood		2	7	.17
CMV in urine		2	4	.08
Smoking		16	34	.07
History of surgery		14	29	.09
Education				
	Illiterate	0	4	.10
	Primary school	4	35	.06
	High school	23	101	.08
	University and higher	9	14	.09

In Nr: number; SEM: standard error of mean.

**Table II:** Comparing the studied variables between participants with and without CMV infection

	**Control**		**Infertility**	
	**Without CMV infection** **(n= 41)**	**With CMV infection** **(n= 20)**	**Without CMV infection** **(n= 134)**	**With CMV infection** **(n= 20)**
Age (years)	35.43 ± 0.98*	31.40 ± 1.029	33.947 ± 0.492	33.40 ± 1.07
BMI (kg/m^2^)	24.02 ± 0.55	23.20 ± 1.68	24.15 ± 0.35	24.75 ± 1.25
Sperm count (* million)	30.09 ± 2.37	33.20 ± 5.73	21.41 ± 1.68	20.35 ± 4.91
Sperm motility (%)	23.48 ± 2.12	28.00 ± 7.45	19.65 ± 1.10	18.65 ± 2.34
Sperm morphology (%)	44.92 ± 2.08	47.20 ± 1.74	15.54 ± 1.25	17.85 ± 3.46

Independent sample t test was employed to compare the studied variables between participants with and without CMV infection,

* p<0.05. Variables are expressed as mean± SEM.

## Discussion

Our findings from patients with and without infertility showed that CMV infection does not have any value in the infertility of our cases. We also showed that there are not any significant differences in sperm count and motility between those with and without CMV infection in cases and controls. Even though that the prevalence of CMV infection is extremely high in Iran, the prevalence of CMV infection was 12.5% in the entire studied population, and was 13% in patients with infertility (9, 10). The findings of the current study question the results on the previous studies of the role of CMV infection in male infertility. The findings of the current study is of great clinical importance as CMV infection is very prevalent in Iran, however we did not find a significant role of this infection in male infertility. Previous studies showing the role of CMV infection in male infertility disorders have shown conflicting results (11, 12).

In consistent with our findings, Yang and collaborators firstly showed that while sero-prevalence and genital tract viral shedding were relatively high in infertile couples in Taiwan, viral shedding did not affect the semen quality. Likewise Eggert *et al* showed that the presence of CMV infection in the genital tract of sub fertile patients is considerable, it does not seem to have a significant role on infertility (5). It is also shown that CMV does not have any impact on the motility and morphology of spermatozoids in the ejaculate. They suggested that even though that CMV seems to be more prevalent in infertile men, it does not have a significant impact on fertility. In another study it was reported that CMV treatment did not improved spermogram after several months. 

Our findings, from a society with a high prevalence of CMV infection showed that it does not have a significant value in the prediction of infertility. On the other hand, there are studies which have shown the contrary. Wu and collaborators showed that the rate of CMV infection is higher among infertile males with pathologic cells in the semen. Spermatogenic cells infected by CMV and HSV-II may cause pathologic lesions and affect spermatogenesis. Morphologically, the infected spermatogenic cells undergo some pathologic alteration, such as apoptosis. However this study suffers from a small samples size. 

In another study by Bezold *et al *the DNA of sexually transmitted pathogens was detected in semen from a high percentage of asymptomatic men with infertility, and was associated with poor semen quality (13). Only 8.7% of patients with infertility had CMV infection in this study. They considered 6 other infections with CMV to come to this conclusion; however they did not report the impact of each infection separately. The principal limitation of the present study was its cross sectional nature which preclude the determination of the direction of causality. However we took advantage of a relatively large sample size and close similarity between groups in most of the potentially confounding variables. 

In conclusion, we did not show a significant role for CMV infection in male infertility. Based on the results, it could be assumed that CMV infection may not have an important role in male infertility. The findings of the present study pave the way for future prospective studies to determine the role of CMV treatment in male infertility.

## References

[1] Hirsh A (2003). Male subfertility. BMJ.

[2] Baker HW (1997). Medical treatment for idiopathic male infertility: is it curative or palliative?. Baillieres Clin Obstet Gynaecol.

[3] DeMasters J (2004). Male factor infertility: an overview of issues. Adv Nurse Pract.

[4] Cooper TG, Noonan E, von Eckardstein S, Auger J, Baker HW, Behre HM (2010). World Health Organization reference values for human semen characteristics. Hum Reprod Update.

[5] Eggert-Kruse W, Reuland M, Johannsen W, Strowitzki T, Schlehofer JR (2009). Cytomegalovirus (CMV) infection-related to male and/or female infertility factors?. Fertil Steril.

[6] Krause W, Herbstreit F, Slenzka W (2002). Are viral infections the cause of leukocytospermia?. Andrologia.

[7] Erles K, Rohde V, Thaele M, Roth S, Edler L, Schlehofer JR (2001). DNA of adeno-associated virus (AAV) in testicular tissue and in abnormal semen samples. Hum Reprod.

[8] Greil AL, Slauson-Blevins K, McQuillan J (2010). The experience of infertility: A review of recent literature. Socio Health Illn.

[9] Behzad-Behbahani A, Entezam M, Mojiri A, Pouransari R, Rahsaz M, Banihashemi M (2008). Incidence of human herpes virus-6 and human cytomegalovirus infections in donated bone marrow and umbilical cord blood hematopoietic stem cells. Indian J Med Microbiol.

[10] Ziyaeyan M, Sabahi F, Alborzi A, Mahboudi F, Kazemnejad A, Ramzi M (2006). Diagnosis and monitoring of human cytomegalovirus infection in bone marrow transplant recipients by quantitative competitive PCR. Exp Clin Transplant.

[11] Nigro G, Mazzocco M, Aragona C, D'Emilio C, Cosmi EV (2000). Hyperimmunoglobulin therapy for cytomegalovirus-associated infertility: case reports. Fertil Steril.

[12] Csata S, Kulcsar G (1991). Virus-host studies in human seminal and mouse testicular cells. Acta Chir Hung.

[13] Bezold G, Politch JA, Kiviat NB, Kuypers JM, Wolff H, Anderson DJ (2007). Prevalence of sexually transmissible pathogens in semen from asymptomatic male infertility patients with and without leukocytospermia. Fertil Steril.

[14] Bezold G, Politch JA, Kiviat NB, Kuypers JM, Wolff H, Anderson DJ (2007). Prevalence of sexually transmissible pathogens in semen from asymptomatic male infertility patients with and without leukocytospermia. Fertil Steril.

